# Oral nerve sheath myxoma: a rare and unusual intraoral neoplasm

**DOI:** 10.1002/ccr3.1341

**Published:** 2017-12-22

**Authors:** Agnieszka M. Frydrych, Norman A. Firth

**Affiliations:** ^1^ Oral Medicine UWA Dental School The University of Western Australia M512, 17 Monash Avenue Nedlands Western Australia 6009 Australia; ^2^ Oral Medicine WA Suite 3, 42‐44 Parliament Place West Perth Western Australia 6005 Australia; ^3^ Oral Medicine and Oral Pathology UWA Dental School The University of Western Australia M512, 17 Monash Avenue Nedlands Western Australia 6009 Australia

**Keywords:** nerve sheath myxoma, neurothekeoma, oral pathology, S100

## Abstract

We present a rare case of intraoral nerve sheath myxoma. Clinically, the neoplasm mimics many other oral mucosal pathosis, underscoring the importance of histopathology in ensuring accurate diagnosis of oral mucosal lesions. Reports of intraoral nerve sheath myxomas are essential to enhance our understanding of this rare intraoral entity.

## Introduction

Nerve sheath myxoma (NSM), also referred to as myxoid neurothekeoma, is an uncommon benign peripheral nerve sheath neoplasm which usually arises within the dermis and subcutaneous tissues of the head and neck and upper extremities 1. The tumor is extremely rare in the oral cavity, typically presenting as a solitary, slow‐growing, asymptomatic, submucosal mass 1, 2. A systematic review published in 2013 identified only 25 clearly documented intraoral cases of NSMs 1. Since that publication, to the best of our knowledge, only two additional cases of intraoral NSM have been published 2, 3. Given the extreme rarity of this intraoral tumor, the aim of this study was to present a case of NSM affecting the tongue in a 43‐year‐old female.

## Case Report

**Figure 1:**
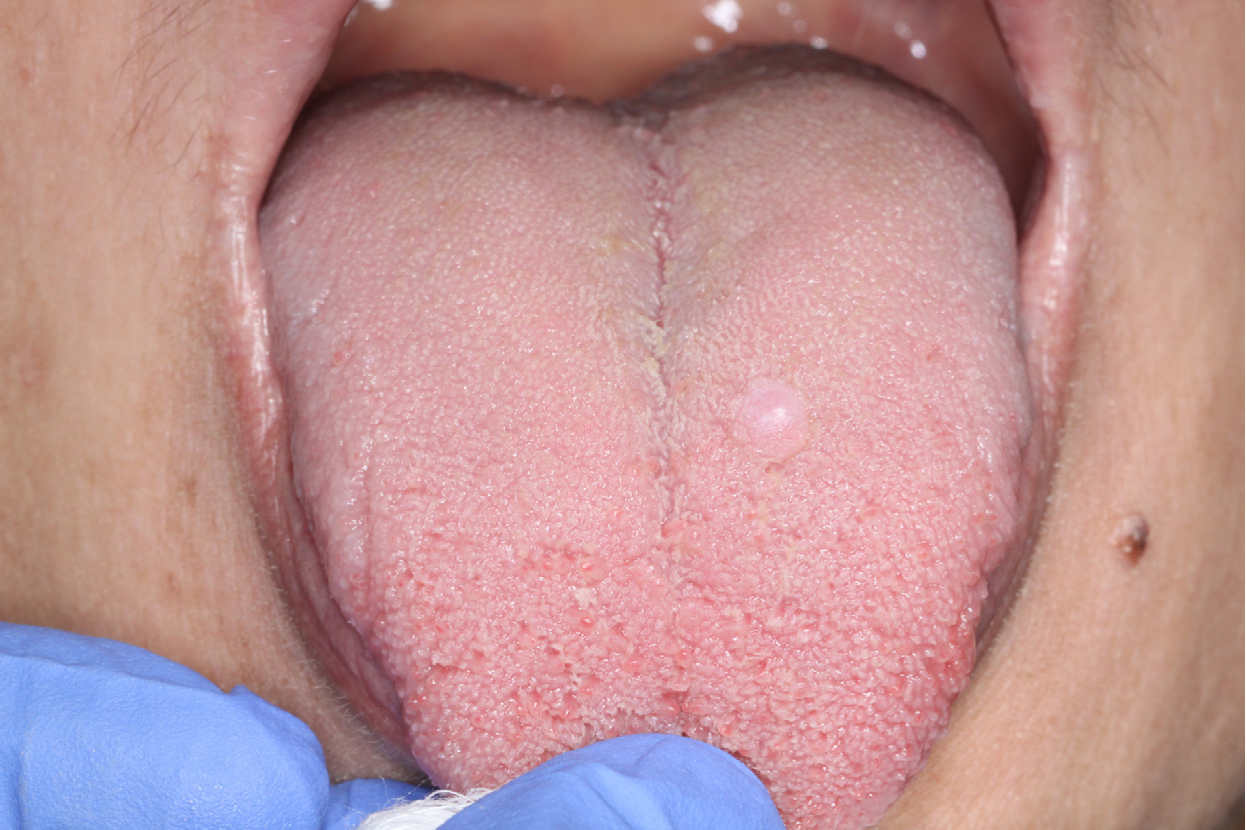
Dorsal surface of tongue showing the lesion at presentation – a small swelling just left of the midline.

A 43‐year‐old female was referred to an oral medicine clinic with regard to a small swelling involving the anterior dorsal surface of her tongue, just left of the midline (Fig. 1). The patient was aware of the presence of the swelling for over 2 years, which was reported to have remained unchanged and asymptomatic, unless bitten. With the exception of a history of depression managed with desvenlafaxine, the medical history was unremarkable. The patient was a nonsmoker and denied any significant alcohol use.

On examination, no extraoral abnormalities were identified and there was no palpable cervical lymphadenopathy. Intraorally, a small sessile mass was noted on the anterior dorsal surface of the tongue, just left of the midline. The lesion was nonulcerated and not tender to palpation. No other oral mucosal abnormalities were identified. Based on the history and clinical examination, a provisional diagnosis of a fibroepithelial polyp was made and an excision was recommended and undertaken.

**Figure 2:**
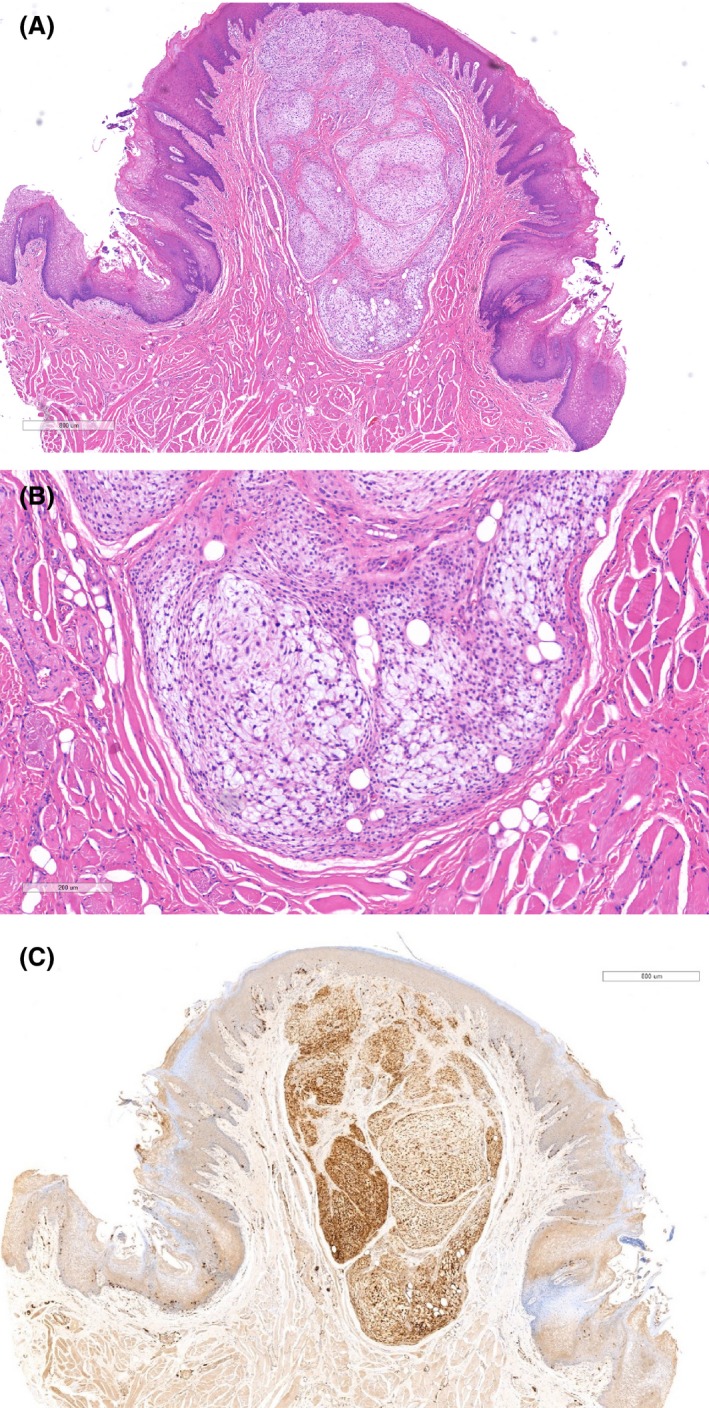
(A) Semipedunculated nonencapsulated, but circumscribed mass covered by stratified squamous epithelium, composed of lobules of fibromyxoid stroma separated by fibrous septae. (B) Within the lobules are numerous randomly distributed stellate and spindle‐shaped cells. Nuclear pleomorphism is not marked and mitotic figures are not conspicuous. (C) Lesional cells exhibit positive immunoreactivity with S100.

**Figure 3:**
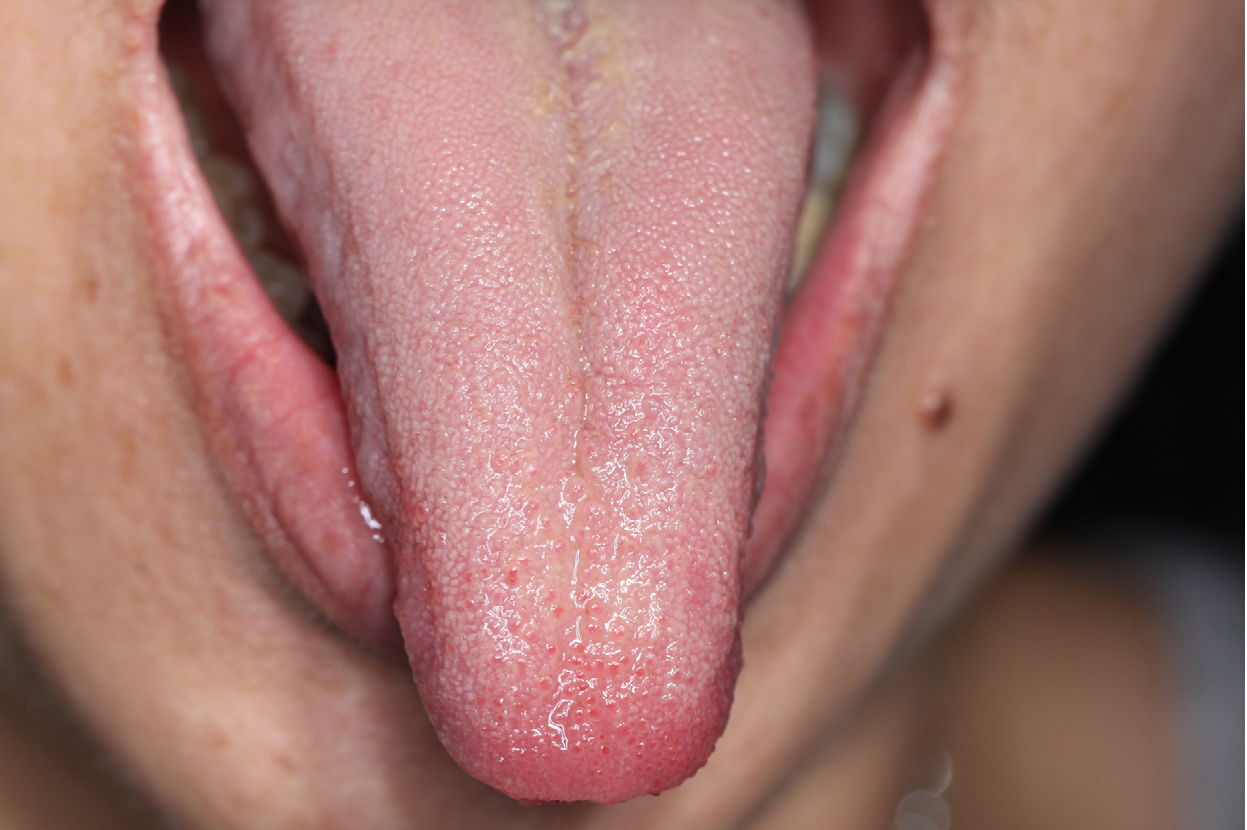
Dorsal surface of tongue showing the absence of tumor recurrence at 18 months.

An ellipse of oral mucosa measuring 10 × 8 × 8 mm was subsequently submitted for histopathological examination. The sections showed a semipedunculated nonencapsulated, but circumscribed mass covered by stratified squamous epithelium (Fig. 2A). Centrally, lobules of fibromyxoid stroma separated by fibrous septae were seen. Within these lobules were numerous randomly distributed stellate and spindle‐shaped cells. Nuclear pleomorphism was not marked and mitotic figures were not conspicuous (Fig. 2B). Lesional cells showed positive immunoreactivity with S100 (Fig. 2C) and vimentin, but were negative for CD34. At these levels, excision appeared complete. A diagnosis of a NSM was established. The patient was reviewed 18 months later with no evidence of recurrence (Fig. 3).

## Discussion

Intraoral NSM is a rare benign tumor, first reported in 1974^4^, with a small additional number of cases reported to date 1, 3, 5, 6, 7, 8, 9, 10, 11, 12, 13, 14. In 1980, Gallager and Helwig^15^ introduced the term neurothekeoma to describe a superficial tumor of presumed nerve sheath derivation which exhibited many clinical and histological similarities to NSM 1. Subsequently, many authors have considered NSM and neurothekeoma as variants of the same tumor, with the NSM constituting the myxoid, or hypocellular type (also called “myxoid neurothekeoma”) and the neurothekeoma the cellular variant 1, 16, 17. Recent evidence, however, suggests that NSMs and neurothekeomas represent distinct entities with different derivations and morphological and in the case of the cellular variant of neurothekeoma, immunohistochemical attributes. Consequently, the use of the terms NSM and neurothekeoma as synonymous or as variants of the same tumor has been strongly discouraged 1. Molecular studies indicate that NSMs are of peripheral nerve sheath origin and suggest that neurothekeoma may be a variant of fibrous histiocytoma 18. Expression of S100 protein indicating a neural origin, differentiates NSM from a cellular neurothekeoma. Unfortunately, the interchangeable use of the terms NSM and neurothekeoma has created a great deal of confusion in the literature regarding these entities 1, 18, 19.

The 2013 systematic review identified the mean age at diagnosis to be 36 years, although NSMs have been reported to occur across the entire age spectrum, from newborns to the elderly (84 years), with a male to female ratio of 1:1.5 1. Gingiva has been reported to represent the most common site of involvement followed by the buccal mucosa, tongue, and other intraoral sites, with the mean lesion duration of 38.2 months prior to diagnosis and with the majority of lesions measuring less than 1 cm 1. A clinical diagnosis of a reactive lesion, that is, a fibroepithelial polyp/traumatic fibroma was the most common 1. This was also true of our patient, who presented with a small, slow‐growing tongue lesion also suspected to represent a fibroepithelial polyp. The fact that many NSMs present as very common oral mucosal pathosis underscores the importance of histopathological examination of all removed lesional tissue, irrespective of its innocuous clinical appearance in order to establish the correct diagnosis.

The etiology of NSM is unknown, although as many occur in areas subject to trauma, it has been proposed that traumatic injury may play a role 1. Aside from fibroepithelial polyps, differential diagnosis may include a mucocele, lipoma, fibrolipoma, schwannoma, and a neurofibroma 6, 8, 12. Histologically, the primary differential diagnosis is a neurothekeoma, which may clinically be indistinguishable from a NSM 1. Nerve sheath myxomas present as well‐circumscribed, nonencapsulated, lobulated lesions, exhibiting proliferation of spindle, stellate, and occasionally epithelioid‐shaped cells in an abundant myxoid stroma 1, 8. Mast cells are frequently seen 1, 6. In general, less cellularity and larger degree of myxomatous change differentiates a NSM from a neurothekeoma, particularly the cellular variant, in which the tumor cells are characterized by hyperchromatic nuclei and high mitotic counts 1, 8. Multinucleated cells are also commonly seen in a neurothekeoma 1, 20. Evaluation of S‐100 protein expression or other neural markers is essential to confirm diagnosis of a NSM 1. Vered et al. reviewing the English literature found that 17 of 19 NSM expressed S‐100 protein and 12 of 13 expressed neuron‐specific enolase 19. Twelve of twelve expressed Vimentin and two of two nerve growth factor receptor 19. They found no cases of positive expression of desmin (*n* = 8), smooth muscle actin (*n* = 5), epithelial membrane antigen (*n* = 4), or AE1/AE3 (*n* = 3) 19. Other histologic differential diagnosis includes plexiform neurofibroma and plexiform schwannoma with myxoid change 1, 7, 10. The histopathological features noted in our case were consistent with a NSM, and diagnosis was confirmed by the positive immunoreactivity with S100.

Nerve sheath myxomas are not known to be associated with any hereditary conditions and have not been reported to undergo malignant transformation 11. Complete excision is both diagnostic and generally curative 1, 10, 12. Recurrence has been attributed to incomplete excision.^8^ No evidence of recurrence was observed in our case, 18 months following the excision.

## Conclusion

Intraoral NSM represents an unusual and extremely rare, benign neoplasm of peripheral nerve sheath origin. The neoplasm mimics other oral mucosal pathosis underscoring the importance of histopathological examination of lesional tissue. Accurate disease description and appropriate use of terminology are essential in ensuring better understanding of rare entities.

## Authorship

AF: was involved in the clinical management and follow‐up of the patient. NF: was responsible for the histopathology. AF and NF: were conjointly responsible for the literature search and preparation of the manuscript. AF: acquired the clinical images, while NF provided the photomicrographs.

## Conflict of Interest

None declared.
